# Microsatellite Instability and Metastatic Colorectal Cancer – A Clinical Perspective

**DOI:** 10.3389/fonc.2022.888181

**Published:** 2022-04-28

**Authors:** Tomas Buchler

**Affiliations:** Department of Oncology, First Faculty of Medicine, Charles University and Thomayer University Hospital, Prague, Czechia

**Keywords:** colorectal cancer, mismatch repair, chemotherapy, nivolumab, pembrolizumab, ipilimumab

## Abstract

Approximately 4-5% of patients with metastatic colorectal cancer (mCRC) have mismatch repair deficient (dMMR)/microsatellite instability-high (MSI-H) tumours. These tumours present challenges in the clinical practice due to variant response to fluoropyrimidine-based chemotherapy and, perhaps, also non-immunologic targeted therapies. Recently, a breakthrough in the treatment of dMMR/MSI-H mCRC has been achieved with several clinical trials showing dramatic long-term benefit of immunotherapy using checkpoint inhibitors. Nevertheless, several questions remain regarding the optimisation of immunotherapy regimens and the use of biomarkers to identify populations set to derive the greatest benefit from immunotherapy. Combination regimens and/or the use of immunotherapy as a maintenance after induction non-immunologic systemic therapy may be the way forward to improve outcomes.

## Introduction

Colorectal cancer (CRC) is one of the main causes of morbidity and mortality, representing approximately 10% of all cancers diagnosed worldwide (1). Mismatch repair deficient (dMMR)/microsatellite instability-high (MSI-H) colorectal tumours are identified in approximately 15% of CRC patients. Due to relatively favourable prognosis in comparison to the mismatch repair proficient (pMMR) CRC, the proportion of these tumours decreases to approximately 4-5% in patients with metastatic CRC (mCRC) (2).

Deficiency in protein products of *MSH2, MLH1* and *MSH6* genes results in impaired detection of mismatched and unpaired bases, leading to the expression of abnormal proteins that may be recognized as neoantigens by the immune system. Additionally, most likely due to Darwinian selection pressure, the deficiency causes failure of apoptosis upon detection of critical DNA damage (2, 3).

## Non-Immunologic Therapies for dMMR/MSI-H CRC

dMMR/MSI-H status has been associated with the lack of benefit from fluoropyrimidines in stage II and III CRC (4–6). Nevertheless, the benefit from oxaliplatin-containing regimens remains unchanged making them the standard of care in patients with CRC requiring adjuvant chemotherapy (6–8). In mCRC the prognosis and response to chemotherapy in patients with dMMR/MSI-H tumours is significantly influenced by the presence of somatic *BRAF* mutations which are associated with poor prognosis (9, 10). *BRAF* V600E mutations are causally linked to *MLH1* promoter hypermethylation leading to deficiency in MLH1 and PMS2 proteins, the most common cause of dMMR/MSI-H phenotype in patients without Lynch syndrome (11–13).

Monoclonal antibodies against the vascular endothelial growth factor (VEGF) or, in patients with *KRAS/NRAS* wild-type tumours, against the epidermal growth factor receptor (EGFR) are commonly added to chemotherapy in mCRC. dMMR/MSI-H patients receiving the VEGF antagonist bevacizumab achieved longer overall survival (OS) compared to patients treated with the EGFR inhibitor cetuximab in the CALGB/SWOG 80405 clinical trial (14). Similar findings were recently reported in a retrospective study (15).

## Immunotherapy for dMMR/MSI-H mCRC

### Pembrolizumab: A Phase 2 Study

Due to the abundance and diversity of neoantigens generated in the absence of functional mismatch repair, immunotherapy using antagonists of the programmed death-1 (PD-1) receptor or its ligand PD-L1), with or without antagonists of the CTLA4 receptor, is an attractive option for patients with dMMR/MSI-H tumours. The pivotal phase 2 study establishing the efficacy of immunotherapy for dMMR/MSI-H tumours was published in 2015 by Le and collaborators and included 10 evaluable patients with colorectal cancer (16). Pembrolizumab was given at a dose of 10 mg/kg biweekly. At 20 weeks, the progression-free survival (PFS) rate in these patients was 78%. The overall response rate (ORR) reached 40% while no responses were seen in patients with pMMR mCRC. In an expanded cohort of 40 patients with mCRC, 2-year PFS and OS were 59 months (95% CI 44 ‐ 78 months) and 72 months respectively (95% confidence interval [CI] 58 ‐ 89 months). Five patients (12%) achieved complete responses (17).

### Nivolumab + Ipilimumab: A Phase 2 Study (CheckMate-142)

CheckMate-142, a nonrandomised multiple cohort phase 2 study, enrolled patients with MSI-H/dMMR as well as pMMR colorectal cancer (18–20). The study comprised several treatment arms including nivolumab monotherapy, nivolumab with ipilimumab, nivolumab with ipilimumab plus cobimetinib (a MEK inhibitor), nivolumab plus relatlimab (a monoclonal antibody against lymphocyte activation gene-3), and nivolumab with daratumumab (a monoclonal antibody against CD38). Data from three cohorts enrolling patients with MSI-H/dMMR mCRC have been published so far.

The first reported cohort included 74 patients with previously treated mCRC (20). However, only 53 of these patients had centrally confirmed dMMR/MSI-H status. The treatment consisted of nivolumab 3 mg/kg every 2 weeks. In the population of 74 patients, PFS at 12 months was 50·4% (95% CI 38·1%–61·4%) and OS was 73·4% (95% CI 61·5%–82·1%). For the subgroups with *BRAF* mutation, *KRAS* mutation, and *BRAFwt/KRASwt*, the ORR was 41.4%, 26.9%, and 25.5%, respectively. The disease control rate was similar in patients with *BRAF* mutation and *BRAFwt/KRASwt*, but lower for patients with *KRAS* mutated tumours (20).

In another cohort of pre-treated patients, patients received nivolumab 3 mg/kg and ipilimumab 1 mg/kg every 3 weeks for four doses followed by biweekly nivolumab 3 mg/kg (18, 19). The majority of patients (76%) had received two or more previous systemic therapies. *BRAF* mutation was present in 25% and *KRAS* mutation in 37% of patients’ tumours. According to 4-year follow-up data, the overall response rate (ORR) reached 65%, with 13% of patients achieving complete response. The ORR was similar in patients with *BRAFwt/KRASwt* tumours and patients with tumours harbouring either mutation. As many as 53% of patients (95% CI 43-62%) were free of progression at 48 months. The OS rate at 48 months was excellent reaching 70.5% (95% CI 61.4-77.9%), far surpassing the expected results for conventional therapy where median OS would fluctuate around 19 months (10).

Finally, results of the cohort receiving nivolumab with ipilimumab as the first-line therapy were published recently (21). Here, 45 patients without previous systemic therapy for mCRC were treated with another combination regimen consisting of nivolumab 3 mg/kg once every 2 weeks and ipilimumab 1 mg/kg once every six weeks until progression or unacceptable toxicity. Interestingly, the 24-month PFS rate was higher for patients with *KRAS* mutated tumours compared to *BRAFwt/KRASwt* tumours, reaching 87.5% (95% CI, 38.7 to 98.1) and 68.4% (95% CI, 35.9 to 86.8), respectively. The 24-month OS rate was 79.4% (95% CI, 64.1 to 88.7).

### Pembrolizumab: A Phase 3 Study (KEYNOTE-177)

KEYNOTE-177 is an ongoing open-label randomised multicentric phase 3 trial for patients without previous systemic treatment for mCRC (22, 23). So far, 307 patients with dMMR/MSI-H mCRC have been randomised to either pembrolizumab (200mg in 3-weekly cycle for a maximum of 35 cycles) or fluoropyrimidine-based chemotherapy with or without non-immunologic targeted agents including bevacizumab or cetuximab. Crossover was allowed for patients in the chemotherapy arm following disease progression. Treatment in the control arm consisted mostly of mFOLFOX6 with bevacizumab (44.8%) or FOLFIRI with bevacizumab (25.2%). Only 11.2% of control arm patients received first-line cetuximab with backbone chemotherapy.

Two-thirds of patients enrolled in the KEYNOTE -177 study had right-sided tumours. As expected, the proportion of patients with *BRAF* V600E mutation in the dMMR/MSI-H population was high (28.1% in the pembrolizumab arm). Unfortunately, molecular analysis data for *KRAS/NRAS* and *BRAF* V600E were not available for 23.8% patients in the study. The median PFS reached 16 months for patients allocated to pembrolizumab (95% CI 5.4-38.1 months) compared to 8.2 months in patients on chemotherapy (95% CI 6.1-10.2 months). The PFS curve seemed to plateau approximately 6 months after the treatment start and about 40% of patients experienced long-term responses. According to a subgroup analysis, patients over 70 years of age did not seem to derive as large benefit from immunotherapy as did younger patients. Of note, patients with *KRAS/NRAS* mutated tumours had numerically superior survival if allocated to the control arm. On the other hand, PFS benefit of immunotherapy was nearly identical for patients with *BRAF* wild-type tumours and those with *BRAF* V600E tumours, although arguably, the control arm regimens were inadequate for this subpopulation and a triplet chemotherapy with bevacizumab should be more effective (24).

The response rate was higher in the pembrolizumab arm with 13.1% patients reaching complete response. Furthermore, responses were long-lasting in the immunotherapy arm with 83.5% of responses ongoing at 24 months.

The final survival analysis was presented at the 2021 American Society of Clinical Oncology (23). OS at 36 months was 61% in the pembrolizumab arm compared to 50% in the control arm (95% hazard ratio [HR] 0.74, 95% CI 0.53-1.03). There was, however, a massive crossover with 60.4% of patients from the control arm eventually receiving anti-PD-1/PD-L1 therapies, and some additional individuals receiving other checkpoint inhibitors (23).

## Biomarkers of Immunotherapy Efficacy in dMMR/MSI-H mCRC

The marked difference in the proportion of dMMR/MSI-H tumours between early and advanced CRC stages points to a strong role of immunoediting in the progression of these tumours. The activation of immunosuppressive pathways linked to PD-1 and CTLA4 protects these tumours from destruction by the immune system and could explain the high efficacy of checkpoint inhibitors in this setting (25). However, only 40-50% of patients with dMMR/MSI-H mCRC derive long-term benefit from the treatment. Possible mechanisms of intrinsic and acquired resistance have been comprehensively reviewed by Sahin et al. (26).

PD-L1 expression was not correlated with benefit from immunotherapy in patients with dMMR/MSI-H mCRC or in the tumour-agnostic trial by Le et al. (16, 18, 19).

The efficacy of checkpoint inhibitors in dMMR/MSI-H patients was not associated with BRAF V600E mutation which is present in approximately 30-40% of dMMR/MSI-H mCRC (19, 23). On the other hand, the apparent lower efficacy of immunotherapy in patients with *KRAS/NRAS* mutated tumours treated with anti-PD-1 monotherapy in the KEYNOTE -177 and CheckMate-142 studies, contrasted with a preserved benefit of combined immunotherapy in CheckMate-142, deserved further study as the subgroups were too small for a valid conclusion (19–21, 23).

Tumour mutation burden (TMB) was a better biomarker for the efficacy of immunotherapy than the absence of an MMR-related protein. It directly correlated with the number of neoantigens (27). Although the great majority of dMMR/MSI-H tumours had high TMB, some dMMR/MSI-H tumours with low TMB have been identified, and they exhibited resistance to therapy with checkpoint inhibitors (28, 29).

Salem et al. compared the TMB according to the type of MMR deficiency. In a heterogeneous cohort of cancers, the largest subgroups comprised endometrial and colorectal tumours. The loss of MSH2/MSH6 was associated with approximately double rate of mutations compared to loss of MLH1/PMS2 (30).

Approximately 3% of colorectal tumours classified as microsatellite stable (MSS) have high TMB, mostly associated with the mutation of polymerase epsilon (*POLE*) or variant *MSH2* (28, 31, 32). These mutations resulted in a high sensitivity to checkpoint inhibitors. Clinical trials enrolling patients with *POLE*-mutated mCRC along those with dMMR/MSI-H mCRC are ongoing (NCT03150706 and NCT03435107, **Table 1**).

**Table 1:** Ongoing clinical trials for dMMR/MSI-H mCRC using immunotherapy.

Study	Tested immunotherapy	Phase	NCT
IBI310 in Combination With Sintilimab in Patients With DNA Mismatch Repair Deficient(dMMR)/Microsatellite Instability High (MSI-H)Locally-advanced or Metastatic Colorectal Cancer	IBI310 (anti-CTLA-4 antibody) Sintilimab (anti-PD-1 antibody)	Phase 2	NCT04258111
A Study of Nivolumab, Nivolumab Plus Ipilimumab, or Investigator’s Choice Chemotherapy for the Treatment of Participants With Deficient Mismatch Repair (dMMR)/Microsatellite Instability High (MSI-H) Metastatic Colorectal Cancer (mCRC) (CheckMate 8HW)	Ipilimumab	Phase 3	NCT04008030
Evaluation of Pembrolizumab (MK-3475) or Co-formulated Pembrolizumab/Quavonlimab (MK-1308A) in Participants With Microsatellite Instability-High (MSI-H) or Mismatch Repair Deficient (dMMR) Stage IV Colorectal Cancer (CRC) (MK-1308A-008)	Pembrolizumab Pembrolizumab/Quavonlimab (anti-CTLA-4 antibody)	Phase 2	NCT04895722
Study of Pembrolizumab (MK-3475) vs Standard Therapy in Participants With Microsatellite Instability-High (MSI-H) or Mismatch Repair Deficient (dMMR) Stage IV Colorectal Carcinoma (MK-3475-177/KEYNOTE-177)	Pembrolizumab	Phase 3	NCT02563002
Avelumab for MSI-H or POLE Mutated Metastatic Colorectal Cancer	Avelumab	Phase 2	NCT03150706
Durvalumab for MSI-H or POLE Mutated Metastatic Colorectal Cancer	Durvalumab	Phase 2	NCT03435107
Combination Chemotherapy, Bevacizumab, and/or Atezolizumab in Treating Patients With Deficient DNA Mismatch Repair Metastatic Colorectal Cancer, the COMMIT Study	Atezolizumab	Phase 3	NCT02997228
PD-1 Antibody Combined With COX Inhibitor in MSI-H/dMMR or High TMB Colorectal Cancer (PCOX)	PD-1 antibody + cox inhibitor	Phase 2	NCT03638297
A Study of Nivolumab Alone or Nivolumab Combination Therapy in Colon Cancer That Has Come Back or Has Spread (CheckMate142)	Ipilimumab Nivolumab	Phase 2	NCT02060188

There is growing evidence that the genomic instability in tumours with mismatch-repair deficiency may result in the formation of gene fusions. Cocco and collaborators found that potentially targetable fusions of oncogenes such as *NTRK*, *BRAF, RET, FGFR*, *ROS1* and A*LK* were present in 5% of dMMR/MSI-H CRC as compared to only 0.4% of MSS CRC cases. Particular enrichment was seen in the subgroup of dMMR/MSI-H *BRAFwt/RASwt* CRC cases with *MLH1* promoter hypermethylation where the occurrence of these aberrations was as high as 42% (13). Vaňkova et al. confirmed these findings identifying oncogenic gene fusions in as many as 9 of 23 colorectal tumours that were dMMR/MSI-H due to *MLH1* promoter hypermethylation and simultaneously *BRAFwt/KRASwt*. The fusions involved *NTRK, ALK* and *BRAF* genes (33). These studies suggest that *BRAFwt/KRASwt* tumours with *MLH1* promoter hypermethylation should also be tested for the presence of oncogene fusions.

It has been shown that dMMR-MSI-H colorectal cancer was associated with specific changes in gut microbiome. *Fusobacterium nucleatum* was enriched in dMMR/MSI-H CRC and associated with lower number of tumour-infiltrating lymphocytes while the opposite has been suggested for MSS colorectal cancer (34). However, somewhat counterintuitively, *F. nucleatum* has been found to be positively associated with response to checkpoint inhibitors, enhancing the expression of PD-1 and PD-L1 and activating the stimulator of interferon genes (STING) signalling (35). The activity of interferon-γ pathway was also associated with response in dMMR/MSI-H CRC in a recent transcriptomics study while VEGF-A expression characterised poor responders (36).

Other putative molecular markers of response to checkpoint inhibitors have been proposed, in particular β2-microglobulin mutations (37). Nevertheless, a recent study failed to corroborate their association with treatment outcomes (38).

Finally, peritoneal involvement manifesting with ascites is associated with resistance to immune checkpoint blockade in dMMR/MSI-H gastrointestinal cancers and markedly inferior patient outcomes, even compared to peritoneal disease without ascites (39).

The search for reliable predictors is ongoing and detailed reviews of potential biomarkers for immunotherapy of colorectal cancer are available (40, 41).

## Conclusions

Immunotherapy is the preferred treatment option for dMMR/MSI-H CRC resulting in long-term treatment responses in approximately 40-50% of patients. As most non-responders will experience progression within 6-8 months of the treatment initiation, the obvious clinical strategies that have been successfully applied in other tumours with similar early treatment characteristics include the combinations of checkpoint inhibitors with chemotherapy and the use of checkpoint inhibitors as a maintenance therapy in non-progressing patients after the induction (42, 43) In both situations, inclusion of a VEGF-targeted therapy in the regimen appears beneficial. Multiple clinical trials are ongoing and our knowledge on the use of immunotherapy in this setting will keep expanding in the following years (**Table 1**).

## Author Contributions

TB has designed and written the manuscript. The author confirms being the sole contributor of this work and has approved it for publication.

## Funding

The work was supported by grant TN-00064190 from the Ministry of Health, Czech Republic to the Thomayer University Hospital. Publication fee was covered by unrestricted grants from Servier, Roche, AstraZeneca, and Bristol Myers Squibb to the First Faculty of Medicine, Charles University, Prague, Czech Republic. The funder was not involved in the study design, collection, analysis, interpretation of data, the writing of this article or the decision to submit it for publication.

## Conflict of Interest

TB received research support and honoraria from Roche, Bristol Myers Squibb, Merck Sharp Dohme, Merck, and AstraZeneca, all unrelated to the present paper.

## Publisher’s Note

All claims expressed in this article are solely those of the authors and do not necessarily represent those of their affiliated organizations, or those of the publisher, the editors and the reviewers. Any product that may be evaluated in this article, or claim that may be made by its manufacturer, is not guaranteed or endorsed by the publisher.
